# Pelvic Floor Disorders and Pelvic Floor Muscle Exercise: A Survey on Knowledge, Attitude, and Practice among Pregnant Women in Northwest Ethiopia

**DOI:** 10.3390/ijerph20054201

**Published:** 2023-02-27

**Authors:** Merete Kolberg Tennfjord, Belayneh Ayanaw Kassie, Zelalem Mengistu Gashaw, Mengstu Melkamu Asaye, Haymanot Alem Muche, Tibebu Tadesse Fenta, Kalkidan Nigussie Chala, Karolina S. Mæland

**Affiliations:** 1 School of Health Sciences, Kristiania University College, 0152 Oslo, Norway; 2 Department of Women’s and Family Health, College of Medicine and Health Science, School of Midwifery, University of Gondar, Gondar P.O. Box 196, Ethiopia; 3 Department of Gynecology and Obstetrics, College of Medicine and Health Science, School of Medicine, University of Gondar, Gondar P.O. Box 196, Ethiopia; 4 Department of Clinical Midwifery, College of Medicine and Health Science, School of Midwifery, University of Gondar, Gondar P.O. Box 196, Ethiopia; 5 University of Gondar Comprehensive Specialized Hospital, Gondar P.O. Box 196, Ethiopia; 6 Department of Physiotherapy, College of Medicine and Health Science, School of Medicine, University of Gondar, Gondar P.O. Box 196, Ethiopia; 7 Faculty of Health and Social Sciences, Western Norway University of Applied Sciences, 5063 Bergen, Norway; 8 Faculty of Health Sciences, SHARE-Center for Resilience in Healthcare, University of Stavanger, 4036 Stavanger, Norway

**Keywords:** pelvic floor dysfunction, pelvic floor muscle exercise, pelvic organ prolapse, urinary incontinence, pregnancy, Ethiopia, women’s health

## Abstract

The purpose of the study was to investigate 1: overall knowledge of pelvic organ prolapse (POP) and urinary incontinence (UI) as well as knowledge, attitudes, and practice of pelvic floor muscle exercise (PFME); and 2: the association of these factors with parity in pregnant women in Gondar, Ethiopia. A facility-based cross-sectional study was performed in the Central Gondar zone, northwest Ethiopia between February and April 2021. The associations between parity and knowledge of POP and UI, and knowledge, attitude, and practice towards PFME were estimated using logistics regression models and presented as crude and adjusted odds ratios with 95% confidence intervals. Nulliparous women were used as the reference. Adjustments were made for maternal age, antenatal care visits, and level of education. The study sample comprised 502 pregnant women: 133 nulliparous, and 369 multiparous. We found no association between parity and knowledge of POP, UI, or knowledge, attitude, and practice toward PFME. The sum score indicated poor knowledge about both POP, UI, and PFME in the study population, and poor attitude and practice of PFME. Despite a high attendance in antenatal care services, knowledge, attitude, and practice were poor, indicating a need for quality improvement of the services.

## 1. Introduction

Pregnancy and childbirth lead to changes in pelvic organ support due to the increasing size and weight of the uterus [1]. Further, childbirth may also serve as a predisposing factor for the development of obstetric pelvic floor disorders (PFD), such as symptoms of urinary incontinence (UI) and pelvic organ prolapse (POP) [2]. Maternity care services are thus key in providing knowledge and skills for the prevention and treatment of such obstetric PFD [3]. Pelvic floor muscle exercise (PFME) is recommended as the first-line prevention and treatment for UI in pregnant and postnatal women [3,4]. Performed during pregnancy, PFME may also have beneficial effects on labor and birth outcomes [3]. PFME performed postpartum may further prevent and/or treat symptoms of POP [3,5], anal incontinence [6], and symptoms of sexual dysfunction [7,8]. No serious adverse effects have been reported after PFME [9]. Further, it is low cost and requires minimal equipment. However, maternity health care providers (nurses, medical doctors including general practitioners, gynecologists/obstetricians), fail to routinely provide pregnant women with intelligible information about obstetric PFD and to counsel PFME [10,11]. A lack of adequate maternal guidance on risk factors, prevention, and treatment options of obstetric PFD has left women with limited knowledge and uncertainty on what to do and how to perform PFME correctly [12]. Further, within the first week postpartum, Vermantel et al. (2015) found that >50% of the new mothers were unable to contract the PFM correctly, and 45% of those who were certain they did a correct PFM contraction, were not [13]. In a study assessing the acceptability and feasibility of antenatal PFME in Nepalese women [14], information on obstetric PFD and proper instruction of PFME by pelvic physiotherapists or trained gynecologists and nurse-midwives were found to be beneficial. However, obstetric PFD and PFME are not routinely addressed in antenatal care (ANC) services in Ethiopia [15]. Further, attendance to ANC services in Ethiopia is generally low (43% having >4 visits), and influenced by maternal residence and educational level [15].

In Ethiopia, the number of women suffering from PFD has been reported >20% [16,17,18]. This number may, however, just be the tip of the iceberg; due to a lack of knowledge of treatment options, embarrassment, and stigmatization, over 2/3 of women with PFD in Ethiopia never consult health professionals regarding their problems [18,19,20]. Further, in a recent study from Gondar, Ethiopia, investigating pregnant women’s knowledge, attitudes, and practice of general antenatal exercises, only 22.6% identified PFME as beneficial for strengthening the pelvic floor muscles (PFM) and only 5.6% practiced these exercises [21]. The latter study did not specifically address knowledge of obstetric PFD and PFME, nor attitudes towards performing PFME.

Creating awareness and improving knowledge of risk factors and treatment options of PFD have shown to be favorable towards attitudes and the practice of PFME among antenatal women in Malaysia [22]. However, women face barriers in terms of motivation and capacity for performing PFME [23,24]. The high prevalence of PFD in Ethiopia, the low overall attendance of ANC, and the low level of women practicing PFME warrant attention. The aims of this study were therefore to investigate: 1. overall knowledge of POP and UI as well as knowledge, attitudes, and practice of PFME; and 2. the association of these factors with parity in pregnant women in Gondar, Ethiopia. We hypothesized that multiparous women would have better knowledge of POP and UI and better knowledge, attitude, and practice of PFME compared to nulliparous women.

## 2. Materials and Methods

This facility-based cross-sectional study was performed in the Central Gondar zone, northwest Ethiopia between February and April 2021, and served as a baseline for an overall community service project. The overall project aimed to improve prevention and the conservative management of obstetric PFD by providing educational and practical classes to pregnant women and health care providers. Methods, definitions, and units conform to the standards jointly recommended by the International Continence Society and the International Urogynecological Association except were specifically noted [25].

### 2.1. Study Sample

Pregnant women attending ANC services in the Central Gondar zone were recruited to participate in this study. The Central Gondar zone contains 85 health centers and 10 hospitals providing health care for nearly 3,000,000 people. Antenatal care is provided as a free-of-cost service in all these facilities. Four districts were in the target area: (i) Gondar Town; (ii) Wogera; (iii) Dembia; and (iv) Gondar Zuria. Among these, seven facilities were randomly selected by lottery method, and pregnant women were recruited based on systematic random sampling with an interval of three. Sample size calculation was performed using Epi info™ version 7. Due to an unknown proportion of obstetric PFD among pregnant women in Gondar, Ethiopia, a 50% proportion was estimated. Thus, the calculated sample was 422 participants, based on a 50% proportion, 10% non-response rate, 5% absolute precision, and 95% confidence interval. However, the project aimed to include as many women as possible beyond the required sample size. Data were collected face-to-face using interviewer-administered questionnaires. Five trained midwives collected the data under the supervision of two trained health professionals. Pregnant women with serious illnesses at the time of data collection were not included in the study (no data available on numbers excluded).

### 2.2. *Data Collection*

The data collection consisted of two parts. The first part covered POP and UI and comprised questions on knowledge of prevention, risk factors, and management of POP (12 questions) and UI (12 questions) [26]. The second part covered PFME and comprised questions on knowledge (15 questions), attitude (8 questions), and practice (4 questions) of PFME [23,27]. The questionnaires were first prepared in English, then translated into Amharic and back to English by members of the research team. To ensure that questions were relevant and understandable, a pre-test was conducted among 10 pregnant women in a health care facility outside the study area, prior to the data collection. Based on comments from the pre-tests, necessary adjustments were made. Socio-demographic factors and obstetric characteristics were included in the questionnaire.

### 2.3. Knowledge about POP and UI

To investigate knowledge of prevalence, risk factors, diagnosis, and treatment of POP and UI, we used the Prolapse and Incontinence Knowledge Questionnaire (PIKQ) [26]. The PIKQ was developed in the United States in 2008 to enable a reliable instrument that assessed patient knowledge about pelvic organ prolapse and urinary incontinence [26]. The validity of the PIKQ has previously been found satisfactory in a gynecologic and uro-gynecologic patient population [26]. The questionnaire contained two distinct 12-item scales: one for POP and one for UI. Each question had three possible responses (Agree/Disagree/Don’t Know). One point was given for each correct answer, giving a maximum of 12 points per scale. To eliminate biases of giving a single similar response in all the items, correct answers varied between *Agree* (15 questions) and *Disagree* (9 questions). Answering *Don’t Know* never gave any points. An aggregate scale score was calculated by summing the number of correct responses. Good knowledge was defined as ≥ 50% correct answers in the PIKQ-POP and ≥80% correct answers for PIKQ-UI.

### 2.4. Knowledge, Attitudes, and Practice of PFME

Questions comprising knowledge, attitudes, and practice of PFME were adopted from previous studies on pregnant women [23,27] that have reported good to very good internal consistency for all sections of the questionnaire. The knowledge section had three possible responses (Yes/No/Don’t Know). One point was given for each correct answer, giving a maximum of 15 points. One question was excluded *“PFME can tighten buttocks muscle”* as the alternative was listed as true [28]. Thus, the knowledge section included 14 items that could give a maximum of 14 points. To eliminate biases of giving a single similar response in all the items, correct answers varied between *Yes* (11 questions) and *No* (3 questions) whereas answering *Don’t Know,* never gave any points. Attitudes towards PFME were assessed with eight Likert scale questions, giving a maximum of eight points. Answering *strongly agree* or *agree* indicated good attitudes towards PFME, resulting in 1 point. Answering *neutral*, *disagree*, or *strongly disagree* indicated poor attitudes towards PFME and resulted in 0 points. To eliminate biases of giving a single similar response in all the items, one of the questions *(I feel that PFME is boring)* indicated poor attitude with reversed answering resulting in 1 point (“disagree”, “strongly disagree”). The practice of PFME was also assessed with four Likert scale questions, giving a maximum of four points. Good practice of PFME was considered when answering *“usually”, “frequently”*, or *“always”* resulting in a score of 1. Poor practice of PFME was considered when answering *“never” or “seldom”*, not resulting in any points. Answers for each question in each section were summarized. Women scoring mean and above were considered to have good knowledge, attitude, and practice of PFME. Women scoring below the mean value were considered to have poor knowledge, attitude, and practice of PFME.

### 2.5. Exposures

The parity of each woman at inclusion was based on the question *“How many times have you delivered before?”* and was used as the exposure in the study. The variable was used as a continuous variable in Table 1 and was dichotomized (nulliparous, multiparous) for the regression analyses.

### 2.6. Background Variables

Maternal residence was based on the question *“Where do you live?”* with two possible options (rural, urban). Maternal age was included as a continuous variable in the analyses. Parity was included as a continuous variable, to estimate mean and standard deviation (SD) of multiparity. Marital status was included as a dichotomous variable (married, unmarried/divorced). Maternal education included four categories (no education/unable to read and write, primary education, secondary education, and ≥college). ANC visits were based on the number of visits during the *present* pregnancy (first time, two times, three times, or ≥four times), corresponding to gestational age, i.e., a woman with long gestational age at the time of the survey had more ANC visits registered (for the present pregnancy) than women with shorter gestational age. Previously heard about POP or UI and PFME was based on the question *“Have you heard about pelvic organ prolapse and/or incontinence?”* and *“Have you heard about pelvic floor muscle exercise?”,* respectively.

### 2.7. Statistical Analyses

Normality tests were performed for all the variables included, maternal age was normally distributed, and remaining variables were right-skewed. Background variables were presented for nulliparous and multiparous separately using mean and SD or frequencies with percentages. Differences in background variables between nulliparous and multiparous women were investigated using Pearson’s Chi-squared test. To investigate possible associations between parity and knowledge of POP and UI, and knowledge, attitude, and practice towards PFME we estimated crude odds ratios (ORs) and adjusted odds ratios (aORs) with 95% confidence intervals (CI) using a logistics regression analysis. For associations between parity and knowledge of POP, nulliparous women with good knowledge of POP (≥ 50% correct answers) and UI (≥80% correct answers) were used as a reference. For associations between parity and good knowledge, attitude, and practice towards PFME, mean scores and above were used as a reference. Adjustments were made for maternal age, ANC visits, and level of education. To assess for multicollinearity, the criteria of Cohen’s d were used (weak association: r = 0.10–0.29; moderate association: r = 0.30–0.49; strong association: r = 0.50 to 1.0). The variables parity, educational level, and ANC visits were weakly associated with each other. However, maternal age showed a strong association with parity. Thus, a stepwise regression using different combinations of the variables was performed. Descriptive analyses were performed for covariates significantly associated with the dependent variables in the regression analysis. Statistical significance was defined as *p* ≤ 0.05. Data were cleaned and coded in Epi Data version 3.1. All analyses were performed using SPSS version 24.0 and STATA version 16 (Stata Statistical Software, College Station, TX, USA).

### 2.8. Ethical Considerations

All women were informed about their ethical and voluntary rights, in that they could withdraw from the study at any time and that they could choose not to answer the questions asked. Before the data collection, a signed consent form was required. The study was approved by the University of Gondar (Community Service Project award number VP/RCS/270). The collected data did not include identifiable information.

The study was conducted in line with the Declaration of Helsinki and approved by the Institutional Review Board of University of Gondar, 15 November 2020 (approval number: VP/RCS/270). Collected data and consent forms were stored according to general data protection regulations. The study followed the Strengthening the Reporting of Observational Studies in Epidemiology [29].

## 3. Results

The total study population included 504 pregnant women attending antenatal care in the Central Gondar zone. Women without registered parity in the survey were excluded from the study (*n* = 2). Thus, the final population was 502 women. Table 1 presents the background characteristics. The study sample comprised 133 (26.4%) nulliparous and 369 multiparous (73.2%) women. Due to a high number of women with two visits at the ANC (68.5%), data indicate that the overall population was in their second stage of pregnancy or later. Nulliparous women were more prone to live in an urban setting (74.2% vs. 56.9%) and had a higher level of education than multiparous women. In total, 49% (*n* = 247) had heard of POP and UI, whereas 26.6% (*n* = 134) had heard of PFME. Further, more multiparous women had heard of POP, UI, and PFME through friends than nulliparous women.

### 3.1. Knowledge about POP and UI

The sum score for knowledge about POP (24.1%) and UI (27%) indicated poor knowledge in the total study population (Table S1). Table 2 presents associations between parity and knowledge of POP and UI for nulliparous and multiparous women. Compared to the multiparous, nulliparous women had a slightly higher proportion of correct answers for both POP and UI (76.7% vs. 74.5% and 74.4% vs. 70.5%, respectively). We found, however, no significant association between parity and knowledge about either POP or UI (Table 2).

When including covariates in the full regression model, we found a linear association between maternal age and good knowledge of POP (aORs 0.93 [95% CI 0.89, 0.97]), but the association was not found for UI (aORs 0.96 [95% CI 0.91, 1.0]). Further, we found a linear association between educational level and better knowledge for both POP and UI (data not shown).

### 3.2. Knowledge, Attitude, and Practice of PFME

The sum score of having good knowledge, good attitude, and good practice towards PFME was 42.0%, 47.2%, and 7.2%, respectively, for the total study population (Table S2).

The association between knowledge, attitude, and practice of PFME with parity is shown in Table 3. Compared to multiparous women, nulliparous women had slightly better knowledge and attitudes about PFME (44.4% and 51.1% versus 41.2% and 45.8%), whereas multiparous women had slightly better practice of PFME than nulliparous women (7.3% versus 6.8%), respectively. There was, however, no significant association between parity and knowledge, attitude, and practice of PFME (Table 3).

In the full model, adjusting for all covariates, no change was seen in the relationship between parity and PFME (Table 3). Only the educational level was significantly associated with having better knowledge of PFME (*p* < 0.001). This relationship showed a linear association between better knowledge of PFME and education, and the educational level explained 20% of the variation of having better knowledge. Having a better attitude was associated with higher maternal age (aORs 0.94 [95%CI 0.90. 0.97]).

## 4. Discussion

Knowledge about POP and UI, and knowledge, attitude, and practice of PFME were considered poor for the pregnant women participating in this study. Further, although nulliparous women showed slightly better knowledge of POP and UI, and slightly better knowledge and attitude for performing PFME, these findings were not significantly associated. These findings oppose our hypothesis, in which we expected that multiparous women would have gained more knowledge related to obstetric PFD and PFME through multiple pregnancies and higher age. There may be several explanations for these findings. First, more multiparous women in our sample had no formal education. Thus, health literacy may be low. Although the regression analysis indicates that higher education is associated with better knowledge of POP, UI, and PFME, these findings are irrespective of parity. Knowledge about postnatal exercises was found to be influenced by parity in a Nigerian study by Mbada et al. [30]. However, the level of education was higher in the latter study as compared to the present, which may be one explanation for these different findings. Second, from our sample, it looks like most pregnant women visited ANC, and the number of visits followed their gestational age, i.e., women with a high gestational age had been to more ANC consultations than women with a lower gestational age. However, with more visits and thus a higher gestational age, there was no difference in knowledge of POP and UI, nor knowledge, attitude, or practice of PFME as compared to those with fewer ANC visits and a lower gestational age.

Salmon et al. (2019) highlighted that women’s uptake of PFME may be constrained by health care providers’ limited attention or women’s poor access to these services [12]. Slightly more multiparous women practiced PFME; these results were however not significantly different from the nulliparous women. A low practice of PFME may follow poor knowledge and attitude and may thus be a reasonable explanation as to why the practice of PFME was no different between multiparous and nulliparous women. Our results are in line with the previous study from Gondar, Ethiopia [21] reporting that less than 6% practiced PFME during pregnancy. The same study also stated that 53.6% of their respondents reported that the practice of ANC exercises (PFME included) was not appropriate in the Ethiopian culture. Results from a previous Nigerian study further point to insufficient knowledge as a factor influencing a negative attitude for performing antenatal exercises [31]. For most women in this present sample, access to a health facility could be within reach since >60% were urban citizens. However, these health facilities may not necessarily include appropriate attention to obstetric PFD or PFME or comprise pelvic physiotherapy services [15]. Moreover, our findings indicate that more women had heard of PFME through media than health professionals, and an equal amount had heard of POP and UI from either media or a health professional. Further, significantly more multiparous women had heard of POP, UI, and PFME through friends than nulliparous women (Table 1). Since the health-related behavior information, such as PFME, seems to be conveyed by non-professionals, this raises a concern related to the quality of the information being put out to these women. This is especially true considering that about 50% of postnatal women are unaware of how to do a proper PFM contraction [13], and a large share of women contract their PFM wrong including external musculature for this exercise [28]. Women seem to lack motivation to practice PFME [23,24], and the lack of a proper assessment and instruction on how to practice PFME may further hamper motivation and success [14,31].

Our results may have looked different if we had included information concerning the presence of PFD. Pregnancy and childbirth may induce obstetric PFDs [1,2], and both parity and age are also risk factors for the development of PFD [2]. Further, according to published data from Ethiopia on the prevalence of PFD [16,18], there are reasons to believe that the pregnant women in our sample would experience these problems. It may be that if women experienced PFD, they may be more motivated to seek information about their problems. However, from a previous study of women with UI, these were no more likely than those with no UI to see a gynecologist or urogynecologist for their problem [32]. Moreover, this lack of consultation with health care providers may be owed to poor knowledge of treatment options available, embarrassment, and stigmatization related to the situation [18,19,20].

The strengths of the study are the relatively large number of pregnant women included and the use of reliable and validated questionnaires to assess knowledge of POP and UI [26] as well as knowledge, attitude, and practice of PFME [23,27]. Another strength is the inclusion of women of different gestational ages, allowing an assessment of the uptake of ANC services throughout pregnancy among pregnant women in Gondar, Ethiopia.

There are some limitations of this study that need to be addressed. Firstly, we have no data on the presence of PFD and may thus not be able to see differences in knowledge, attitude, and practice of PFME among those with and without these problems. Second, due to its cross-sectional design, we cannot address causal factors for our findings. Third, our study may be underpowered to assess associations of knowledge of POP and UI, and knowledge, attitude, and practice of PFME, and thus be the reason for the non-significant associations with parity. Lastly, although the sampling of participants aimed for a random selection of pregnant women in Gondar, Ethiopia, we may not necessarily be able to generalize our findings to other pregnant women in Ethiopia.

The result of this study illuminates a severe knowledge gap of obstetric PFD for pregnant women in the Gondar zone, Ethiopia, despite a high attendance to ANC services among these women. More information on POP and UI is important, as well as precise information and education on PFME for pregnant women in the region of Gondar. Creating awareness and improving knowledge of risk factors and treatment options of obstetric PFD comprise an important task for maternal health care providers, and results from this study may be used as a guide which focuses on areas of maternal education pre-, per-, and postpartum that need to be improved. Improving the quality of the service including qualified health care personnel such as pelvic physiotherapists that could undertake a proper assessment of the PFM and instruction on how to practice PFME could therefore be an important part of this work and thus secure women with adequate knowledge about obstetric PFD and PFME. It is highly likely that pregnant women in other parts of Ethiopia would benefit from the same information education through ANC, especially in areas of limited access to these health care services. Further, studies on the feasibility and effectiveness of PFME to prevent and treat obstetric PFD are of importance. The latter could be carried out through ANC services, as our results illustrate an opportunity to gather pregnant women in this period of life.

## 5. Conclusions

The results from the present study show that pregnant women in Gondar, Ethiopia have poor knowledge of POP and UI, and poor knowledge, attitudes, and practices of PFME. No association was found for parity and the above factors. Most pregnant women utilized ANC services throughout their pregnancy. However, despite a high attendance in ANC, knowledge was poor, as well as attitude and practice, indicating a need for quality improvement of the services.

## Figures and Tables

**Table 1:** Background characteristics for the study population by maternal parity, *n* = 502.

	Total Population	Nulliparous	Multiparous	*p*-Value ^a^
**No. of women** **(%)**	502 (100)	133 (26.5)	369 (73.5)	
**Residence** ***n*** **(%)**				<0.001
Urban	308 (61.5)	98 (74.2)	210 (56.9)	
Rural	193 (38.5)	34 (25.8)	159 (43.1)	
**Maternal age** (mean ± SD)	28.1 ± 6.2	23.4 ± 4.0	29.8 ± 5.9	<0.001
**Parity** (mean ± SD)	1.8 ± 1.8	0 ± 0	2.5 ± 1.6	<0.001
**Married** ***n*** **(%)**	428 (85.3)	110 (82.7)	318 (86.2)	0.30
**Maternal education** ***n*** **(%)**				<0.001
No education (unable to read and write)	168 (33.5)	23 (17.3)	145 (39.3)	
Primary education	110 (21.9)	25 (18.8)	85 (23.0)	
Secondary education	87 (17.3)	42 (31.6)	45 (12.2)	
Collage and above	137 (27.3)	43 (32.3)	94 (25.5)	
**Antenatal care visits *n* (%)**				0.02
First visit	158 (31.5)	50 (37.6)	108 (29.3)	
Second visit	156 (31.0)	32 (24.1)	124 (33.6)	
Third visit	105 (20.9)	22 (16.5)	83 (22.5)	
Fourth visit or more	83 (16.5)	29 (21.8)	54 (14.6)	
**Previously heard about pelvic organ prolapse/** **urinary incontinence** ^b^ ***n*** **(%)**				
Yes	247 (49.2)	68 (51.1)	179 (48.5)	0.60
*From health care provider*	*110 (44.5)*	*30 (44.1)*	*80 (44.7)*	0.94
*From family*	*40 (16.2)*	*10 (14.7)*	*30 (16.8)*	0.70
*From friends*	*59 (23.9)*	*10 (14.7)*	*49 (27.4)*	0.04
*From media*	*111 (45.1)*	*36 (52.9)*	*75 (42.1)*	0.13
**Previously heard about pelvic floor muscle exercise** ^b^ ***n*** **(%)**				
Yes	134 (26.5)	43 (32.3)	90 (24.4)	0.08
*From health care provider*	53 (40.2)	19 (44.2)	34 (38.2)	0.51
*From family*	11 (8.3)	4 (9.3)	7 (7.9)	0.78
*From friends*	38 (28.8)	5 (11.6)	33 (37.1)	0.002
*From media*	84 (64.1)	31 (72.1)	53 (60.2)	0.18

Reported as numbers (*n*) with percentages (%) or means with standard deviation (SD); ^a^ calculated as the difference between nulliparous and multiparous; ^b^ multiple answers possible.

**Table 2:** Associations between knowledge of pelvic organ prolapse, urinary incontinence, and parity for pregnant women in Gondar, Ethiopia. *n* = 502.

Parity	Nulliparous *n* = 133 (26.4%)	Multiparous *n* = 369 (73.2%)	Crude ORs (95% CI) ^a^	Adjusted ORs (95% CI) ^a,b^
**Pelvic organ prolapse *n* (%)**				
Good knowledge ^c^ (*n* = 377)	102 (76.7)	275 (74.5)	1 (Reference)	1 (Reference)
Poor knowledge (*n* = 125)	31 (23.3)	94 (25.5)	1.1 (0.7, 1.8)	0.8 (0.4, 1.5)
**Urinary incontinence, *n* (%)**				
Good knowledge ^c^ (*n*= 359)	99 (74.4)	260 (70.5)	1 (Reference)	1 (Reference)
Poor knowledge (*n* = 143)	34 (25.6)	109 (29.5)	1.2 (0.8, 1.9)	0.9 (0.5, 1.6)

^a^ Nulliparous women were used as reference category for all analyses; ^b^ adjusted for maternal age, antenatal care visits, and education; ^c^ good knowledge was defined as ≥ 50% correct answers in the *Prolapse and Incontinence Knowledge Questionnaire for pelvic organ prolapse*, and ≥80% correct answers in the *Prolapse and Incontinence Knowledge Questionnaire for urinary incontinence*. Logistic regression models with associations presented with odds ratios (ORs) with 95% confidence intervals (CI) were undertaken.

**Table 3:** Associations between knowledge, attitude, and practice towards pelvic floor muscle exercise and parity for pregnant women in Gondar, Ethiopia. *n* = 502.

	Nulliparous *n* = 133 (26.4%)	Multiparous *n* = 369 (73.2%)	Crude OR (95%CI) ^a^	Adjusted OR (95%CI) ^a,b^
**Knowledge**				
Good knowledge ^c^ (*n* = 211)	59 (44.4)	152 (41.2)	1 (reference)	1 (reference)
Poor knowledge (*n* = 291)	74 (55.6)	217 (58.8)	1.14 (0.76, 1.70)	0.62 (0.37, 1.04)
**Attitude**				
Good knowledge ^c^ (*n* = 237)	68 (51.1)	169 (45.8)	1 (reference)	1 (reference)
Poor knowledge (*n* = 265)	65 (48.9)	200 (54.2)	1.24 (0.83, 1.84)	1.34 (0.81, 2.21)
**Practice**				
Good (*n* = 36)	9 (6.8)	27 (7.3)	1 (reference)	1 (reference)
Poor (*n* = 466)	124 (93.2)	342 (92.7)	0.92 (0.42. 2.01)	0.84 (0.34, 2.07)

^a^ Nulliparous women were used as reference category for all analyses; ^b^ adjusted for maternal age, antenatal care visits, and education; ^c^ good knowledge, attitude, and practice of pelvic floor muscle exercise are summarized as answers mean and above, whereas poor knowledge is answers summarized as below the mean. Differences between good or poor on parity (nulliparous and multiparous) were assessed using the Chi-squared test and presented with frequencies (*n*) and percentages (%). Logistic regression models with associations presented with odds ratios (ORs) with 95% confidence intervals (CI) were undertaken.

## Data Availability

The data presented in this study are available on request from the corresponding author.

## Supplementary Materials

The following supporting information can be downloaded at: https://www.mdpi.com/article/10.3390/ijerph20054201/s1, Table S1: Sum score for knowledge on pelvic organ prolapse and urinary incontinence. Table S2: Sum score for knowledge on pelvic floor muscle exercise.
