# Supplementary material for: Pelvic Floor Disorders and Pelvic Floor Muscle Exercise: A Survey on Knowledge, Attitude, and Practice among Pregnant Women in Northwest Ethiopia
Source: Int J Environ Res Public Health. 2023 Feb 27;20(5):4201. doi: 10.3390/ijerph20054201 (PMC10001623; doi:10.3390/ijerph20054201)
Supplement: Supplementary file 1 [file ijerph-20-04201-s001.zip › ijerph-2230101-supplementary.pdf]

Table S1. Knowledge on pelvic organ prolapse and urinary incontinence

Q no	Question	Agree	Disagree	Don't know	Score
	**Total number of women:** 502	**No. of women,** n (%)			
**300a**	**Previously heard about pelvic organ prolapse / urinary incontinence** (%)	247 (49.2)	255 (50.8)	0 (0.0)	247 (49.2)
	**Pelvic organ prolapse (50%)**				
**301**	**Pelvic organ prolapse is more common in young women than old women**	99 (19.6)	158 (31.4)	247 (49.0)	158 (31.5)
**302**	**Giving birth many times may lead to pelvic organ prolapse.**	191 (37.9)	91 (18.1)	222 (44.1)	191 (38.1)
**303**	**Pelvic organ prolapse can happen at any age.**	157 (31.2)	94 (18.7)	253 (50.2)	157 (31.3)
**304**	**Certain exercises can help to stop pelvic organ prolapse from getting worse.**	140 (27.8)	77 (15.3)	287 (56.9)	140 (27.9)
**305**	**Symptoms of pelvic organ prolapse may include pelvic heaviness and/or pressure.**	139 (27.6)	48 (9.52)	317 (62.9)	139 (27.7)
**306**	**A good way for a doctor to diagnose pelvic organ prolapse is by examining the patient.**	235 (46.6)	41 (8.13)	228 (45.2)	235 (46.8)
**307**	**Once a patient has pelvic organ prolapse, not much can be done to help her**	74 (14.7)	193 (38.3)	237 (47.0)	193 (38.5)
**308**	**Heavy lifting on a daily basis can lead to pelvic organ prolapse.**	250 (49.6)	62 (12.3)	192 (38.1)	250 (49.8)
**309**	**Surgery is one type of treatment for pelvic organ prolapse.**	241 (47.8)	52 (10.3)	211 (41.9)	241 (48.0)
**310**	**Doctors can run a blood test to diagnose pelvic organ prolapse**	215 (42.7)	44 (8.73)	245 (48.6)	44 (21.8)
**311**	**A rubber ring called a pessary can be used to treat symptoms of pelvic organ prolapse.**	74 (14.7)	58 (11.5)	372 (73.8)	74 (14.7)
**312**	**People who are obese are less likely to get pelvic organ prolapse.**	82 (16.3)	58 (11.5)	372 (73.8)	58 (11.6)
	**Good knowledge (%)**				**1,880 (24,1)**

Q no	Question	Agree	Disagree	Don't know	Score
	**Total no of women:** 502	**No. of women,** n (%)			
	**Urinary incontinence (80%)**				
**313**	**Urinary incontinence is more common in young women than in old women.**	249 (49.4)	58 (11.5)	197 (39.1)	58 (11.6)
**314**	**Women are more likely than men to leak urine.**	218 (43.3)	60 (11.9)	226 (44.8)	218 (43.4)
**315**	**Other than pads and diapers, not much can be done to treated leakage of urine**	65 (12.9)	173 (34.3)	266 (52.8)	173 (34.5)
**316**	**It is NOT important to diagnose the type of urine leakage before trying to treat it.**	112 (22.2)	167 (33.1)	225 (44.6)	167 (33.3)
**317**	**Many things can cause urine leakage.**	202 (40.1)	45 (8.93)	257 (51.0)	202 (40.2)
**318**	**Certain exercises can be done to help to control urine leakage.**	145 (28.8)	76 (15.1)	283 (56.2)	145 (28.9)
**319**	**Some medications may cause urinary leakage.**	145 (28.2)	72 (14.3)	287 (56.9)	145 (28.9)
**320**	**Once people start to leak urine, they are never able to control their urine again**	83 (16.5)	169 (33.6)	251 (49.9)	169 (33.7)
**321**	**Doctors can do special types of bladder testing to diagnose urine leakage**	249 (49.4)	42 (8.33)	213 (42.3)	249 (49.6)
**322**	**Surgery is the only treatment for urinary leakage**	129 (25.6)	113 (22.4)	262 (52.0)	113 (22.5)
**323**	**Giving birth many times may lead to urine leakage.**	203 (40.3)	84 (16.7)	217 (43.1)	203 (40.4)
**324**	**Most people who leak urine can be cured or improved with some kind of treatment**	268 (53.2)	33 (6.55)	203 (40.3)	268 (53.4)
	**Good knowledge (%)**				**2,110 (27.0)**

N = number; % = percentages. One point was given for each correct response, no points were given for incorrect responses, blank responses, or for responding *"I don't know"*. Proficiency was defined as scores of 80% or more on the urinary incontinence-scale, and 50% or more on the pelvic organ prolapse-scale. Scores were summarized for each question. The total score was then added and divided by the total study sample. Good knowledge was calculated by all correct answers divided by the study sample*number of questions.

Table S2. Knowledge, attitude and practice of pelvic floor muscle exercise

Q no	Question	False	True	Don't know			Score
	**Total number of women:** 502	**No. of women,** n (%)					
	**Knowledge towards Pelvic floor muscle exercise**						
**401**	**The PFM are situated at the pubic region**	82 (16.3%)	113 (22.4%)	309 (61.3%)			113 (22.4)
**402**	**The PFM involves muscles at the anal region**	61 (12.1%)	124 (24.6%)	319 (63.3%)			124 (24.6)
**403**	**Muscles surrounding the vagina are not involved in the PFM**	46 (9.1%)	140 (27.8%)	318 (63.1%)			46 (9.1)
**404**	**The PFM are important in controlling bladder function**	40 (7.9%)	177 (35.1%)	287 (56.9%)			177 (35.1)
**405**	**The PFM are not involved in controlling the leakage of stool and gas from the anus**	110 (21.8%)	93 (18.5%)	301 (59.7%)			110 (21.8)
**406**	**The PFM are not involved in tightening the vagina**	95 (18.8%)	94 (18.7%)	315 (62.5%)			95 (18.8)
**407**	**The PFM is important in sexual activity**	46 (9.1%)	168 (33.3%)	290 (57.5)			168 (33.3)
**408**	**PFME can tighten buttocks muscles**[a]	Missing	Missing	Missing			Missing
**409**	**PFME can prevent UI during laughing/sneezing/weight bearing**	52 (10.3%)	159 (31.5%)	293 (58.1%)			159 (31.5)
**410**	**PFME can prevent/treat uterine prolapse**	50 (9.9%)	151 (30%)	303 (60.1%)			151 (30)
**411**	**PFME can be done at any time during a life span**	60 (11.9%)	125 (24.8%)	319 (63.3%)			125 (24.8)
**412**	**PFME can be done while performing daily activities**	53 (10.5%)	137 (27.2%)	314 (62.3%)			137 (27.2)
**413**	**Muscles involved should be contracted for 8 seconds**	49 (9.7%)	62 (12.3%)	393 (78%)			62 (12.3)
**414**	**The PFM should be contracted 8–10 times per exercise/round**	41 (8.1%)	60 (11.9%)	403 (80%)			60 (11.9)

**415**	**PFME should be done at least 3 times a day (morning, midday and afternoon)**	40 (7.9%)	106 (21%)	358 (71%)			106 (21)
	**Good knowledge (%)**						**42.0**
**Q no**	**Question**	**Strongly Disagree**	**Disagree**	**Neutral**	**Agree**	**Strongly agree**	**Score**
	**Total number of women:** 502	**No. of women,** n (%)					
	**Attitude towards Pelvic floor muscle exercise**						
**416**	**PFME should be done by all women**	44 (8.7%)	71 (14.1%)	189 (27.5%)	156 (31%)	44 (8.7%)	200 (39.7)
**417**	**I should practice PFME to prevent/treat UI**	57 (11.3%)	88 (17.5%)	177 (35.1%)	147 (29.2%)	35 (6.9%)	182 (36.1)
**418**	**I should practice PFME to prevent uterine prolapse**	53 (10.5%)	75 (14.9%)	188 (37.3%)	149 (29.6%)	39 (7.7%)	188 (37.3)
**419**	**I feel that PFME is boring**	49 (9.7%)	79 (15.7%)	196 (38.9%)	148 (29.4%)	32 (6.3%)	128 (25.4)
**420**	**PFME should be taught to all antenatal mothers at antenatal clinics**	53 (10.5%)	61 (12.1%)	190 (37.7%)	154 (30.6%)	46 (9.1%)	200 (39.7)
**421**	**I support those who want to perform PFME**	42 (8.3%)	61 (12.1%)	199 (39.5%)	152 (30.2%)	50 (9.9%)	202 (40.1)
**422**	**I view that PFME can increase sexual satisfaction**	49 (9.7%)	93 (18.5%)	230 (45.6%)	105 (20.8%)	27 (5.4%)	132 (26.2)
**423**	**I will put in effort to search for info about PFME**	39 (7.7%)	77 (15.3%)	194 (38.5%)	159 (31.5%)	35 (6.9%)	194 (38.5)
	**Good attitude (%)**						**47.2**

Q no	Question	Never	Seldom	Usually	Frequently	Always	Score
	**Total number of women:** 502	**No. of women,** n (%)					
	**Practice towards Pelvic floor muscle exercise**						
**424**	**I have performed PFME when not pregnant**	464 (92.1%)	27 (5.4%)	8 (1.6%)	4 (0.8%)	1 (0.2%)	13 (2.6)
**425**	**I have spent time to perform PFME**	463 (91.9%)	29 (5.8%)	8 (1.6%)	3 (0.6%)	1 (0.2%)	12 (2.4)
**426**	**I have discussed PFME with friends**	458 (90.9%)	29 (5.8%)	7 (1.4%)	7 (1.4%)	3 (0.6%)	17 (3.4)
**427**	**I have tried to search for info about PFME**	445 (88.3%)	36 (7.1%)	13 (2.6%)	8 (0.4%)	2 (0.4%)	23 (4.6)
	**Good practice (%)**						**7.2**

[a]Missing question; PFME=pelvic floor muscle exercise; n=number; %=percentages. Scores were summarized for each question in addition to a total score for each category (knowledge, attitude and practice of PFME). The total score was then dichotomized with scores being mean and above indicating good knowledge, attitude and practice for PFMT and scores below the mean indicating poor knowledge, attitude and practice for PFME.
